# Hypertension management in older adults

**DOI:** 10.12688/f1000research.20323.1

**Published:** 2020-08-19

**Authors:** Ozlem Bilen, Nanette K. Wenger

**Affiliations:** 1 Division of Cardiology, Department of Medicine, Emory University School of Medicine, Atlanta, GA, USA; 2 Emory Heart and Vascular Center, Atlanta, GA, USA; 3 Emory Women’s Heart Center, Atlanta, GA, USA

**Keywords:** hypertension, elderly, blood pressure

## Abstract

Vascular aging leads to arterial hypertension, which is the leading cause of cardiovascular mortality and morbidity in older adults. Blood pressure reduction is effective in reducing the cardiovascular risk and is safe in ambulatory older adults. It is important to note that blood pressure control in this group of patients is challenging because of comorbidities, polypharmacy, and frailty. Choice of pharmacotherapy is not simple and should be individualized.

## What are the burden and mechanism of hypertension in older adults?

Vascular aging involves endothelial dysfunction and vascular remodeling. This process leads to an increase in large artery stiffness and isolated systolic hypertension, the predominant form of hypertension in older adults ^1, 2^, affecting more than 75% of people older than 75 years in the US ^2^.

Hypertension is not a benign age-related phenomenon and indeed remains the leading cause of preventable cardiovascular mortality and morbidity ^3– 6^. The number of older patients continues to increase dramatically to be 8.5% of the world’s current population and this percentage is expected to reach about 17% by 2050 ^7^. Hence, appropriate management of hypertension in this vulnerable population is crucial.

## Do older adults benefit from blood pressure reduction?

Several randomized controlled trials have demonstrated that blood pressure (BP) lowering in systolic hypertension at an older age is effective in reducing the risk of fatal and non-fatal stroke, cardiovascular events, and death ^8– 16^.

One of the few interventions shown to reduce mortality risk in older individuals is BP reduction. One of the earlier hypertension trials in this group of individuals was the Hypertension in the Very Elderly Trial (HYVET). Individuals older than 80 years with an initial systolic BP (SBP) greater than 160 mm Hg were assigned to receive either treatment or placebo ^10^. Reduction of BP significantly decreased the incidence of fatal stroke, all-cause mortality, any cardiovascular event, and heart failure. Another important study that included older individuals was the Systolic Blood Pressure Intervention Trial (SPRINT). Patients older than 50 years with SBP greater than 130 mm Hg and at least one additional cardiovascular risk factor (presence of clinical or subclinical cardiovascular disease other than stroke, chronic kidney disease, a Framingham Risk Score for 10-year cardiovascular disease risk of at least 15, or age greater than 75 years) were enrolled. Patients were randomly assigned to intensive SBP lowering (<120 mm Hg) or routine SBP management (<140 mm Hg). The trial was concluded early because of overwhelming evidence of benefit. There was significant reduction of the primary outcome (myocardial infarction, acute coronary syndrome, stroke, congestive heart failure, or cardiovascular death) (5.2% versus 6.8%, hazard ratio [HR] 0.75, 95% confidence interval [CI] 0.64–0.89; *P* <0.0001). Among patients at least 75 years of age (n = 2,636), primary outcomes for intensive versus routine BP management were 7.7% versus 11.2% ( *P* <0.05). Rates of all-cause mortality were 5.5% versus 8.1%, respectively ( *P* <0.05). There was a significantly increased risk of hypotensive events and metabolic derangements such as hyponatremia with intensive treatment. It is important to note that generalizability of results was limited as patients with diabetes mellitus, history of stroke, and heart failure within 6 months and residents of nursing homes were excluded and less than 40% of patients were female ^17^. The SPRINT trial not only showed the clear benefit of hypertension treatment at an older age but also brought a new perspective to the intensive BP control controversy in this vulnerable population.

Another relevant clinical question when it comes to treating hypertension in older individuals is whether it is beneficial for cognitive function. Longitudinal observational data have shown a strong association between elevated BP and cognitive decline later in life ^18^; indeed, midlife hypertension is considered to be a major risk factor for dementia ^19^.

Whereas several trials demonstrated a lower incidence of dementia with anti-hypertensive treatment in older individuals ^9, 20– 22^, others failed to show benefit and this was due in part to inadequate duration for follow-up ^23– 25^. A recently published sub-study of the SPRINT trial (SPRINT-MIND) did not show a significantly reduced risk of probable dementia with intensive BP control ^26^. The study was terminated early and thus the cases of dementia were fewer than expected, and the study may have been underpowered for this endpoint. However, intensive BP control did significantly reduce the risk of mild cognitive impairment (14.6 versus 18.3 cases per 1000 person-years; HR 0.81, 95% CI 0.69–0.95) and the combined rate of mild cognitive impairment or probable dementia (20.2 versus 24.1 cases per 1000 person-years; HR 0.85, 95% CI 0.74–0.97), which were predefined secondary endpoints.

## What is the optimal target blood pressure in older adults?

Although the benefit of hypertension treatment in older adults has been clearly demonstrated, several cross-sectional studies have raised questions about the risk of hypotension and related cerebrovascular accidents, falls, and kidney failure ^27, 28^; thus, the safety of BP reduction at an older age has been a controversial topic.

Recent US guidelines recommended initiation of anti-hypertensive drug therapy in older individuals with a BP greater than 130/80 mm Hg ^29^, although the BP target for treatment differed in European guidelines ^30^. This recommendation was based on several anti-hypertensive therapy trials that included large numbers of older individuals ^31– 34^. Some patients with mild frailty were also included but most of these patients were ambulatory and able to travel to a clinic. In these trials, cardiovascular morbidity and mortality were reduced during BP lowering with different BP targets, including when the SBP treatment goal was less than 120 mm Hg. However, the risk of adverse outcomes, including orthostatic hypotension or falls, did not increase ^9, 10, 12, 35^. Both HYVET and SPRINT included patients who were frail but still living independently in the community, and both were stopped early for benefit.

Thus, it is fair to say that BP control in ambulatory older patients seems to be safe. However, it is important to recognize that evidence-based recommendations for BP management are lacking in those who are very frail or institutionalized or those with cognitive impairment, several comorbidities, or polypharmacy. There is no evidence in the latter group that anti-hypertensive treatment reduces cardiovascular events or promotes established cognitive dysfunction ^36^, and evidence suggests that such treatment may not be safe ^37^. These individuals might be at risk for adverse consequences; thus, it is important for the clinician to pursue an individualized approach. Clinicians should carefully titrate BP lowering in persons with a high comorbidity burden ^37– 39^.

Another important consideration is initiation of anti-hypertensive therapy with two agents. These individuals need to be monitored carefully for orthostatic hypotension and history of falls. BP must be measured both sitting and standing. Older persons may present with neurogenic orthostatic hypotension. This is particularly common in neurodegenerative disorders such as Parkinson’s disease and hence should be taken into consideration.

## What are the pharmacologic options for hypertension management in older adults?

Lifestyle measures are important in addition to pharmacotherapy. These include sodium restriction—Dietary Approaches to Stop Hypertension (DASH) diet—and physical activity ^40^. BP control in older adults is challenging because of the presence of other comorbidities, polypharmacy, and frailty. Physicians should individualize management to weigh the benefits and potential harmful effects of such therapy.

Common anti-hypertensive drugs, including diuretics, angiotensin-converting enzyme (ACE) inhibitors, calcium channel blockers, and even beta-blockers, have been shown to be effective in older adults ^41– 44^.

It is important to note that each commonly used drug class has additional benefit in the presence of different comorbidities, such as ACE inhibitors in diabetes mellitus and beta-blockers in coronary disease. Decisions should be based on efficacy, tolerability, cost-effectiveness of each drug, and the presence of specific comorbidities and polypharmacy and thus drug interactions.

Beta-blockers should be used cautiously in older patients as these individuals are particularly sensitive to bradycardic effects. The main adverse effects of calcium channel blockers are related to edema and orthostatic hypotension. Also, constipation might be a significant limitation with diltiazem and verapamil. Nitrates are useful during hypertensive emergencies and angina; however, they are not preferred for long-term BP control at an older age. Owing to their side effect profile, peripheral agents such as hydralazine and minoxidil are not first-line agents. A centrally acting agent, clonidine, can be used for BP management. Owing to reflex hypertension when discontinued, oral clonidine is not advised; clonidine patch averts this problem. It has several side effects, including bradycardia and sedation, both of which are problematic in older and frail individuals. Diuretics should be used with caution as urinary incontinence can be a major concern in older adults.

Another important consideration in the management of hypertension in older individuals is to use ambulatory BP monitoring instead of relying on single office measurements. The development of isolated systolic hypertension (and thus wide pulse pressure), white coat effect, different measurements between ambulatory and clinical BP, prevalence of orthostatic hypotension, and medication interactions are among the reasons that necessitate ambulatory BP monitoring ^45^.

Finally, another important consideration is de-escalation of anti-hypertensive medications in patients who are intolerant. The Discontinuation of Anti-hypertensive Treatment in Elderly people (DANTE) study showed that in older individuals with orthostatic hypotension, discontinuation of anti-hypertensives showed significant recovery from orthostasis ^46^. Discontinuation of therapy might also be considered in older patients approaching the end of life, in whom the burden of therapy might exceed the purported benefit.

## What are the future considerations?

Randomized clinical trials are needed in frail older individuals with hypertension residing in nursing homes to determine both the benefits and adverse effects of anti-hypertensive drug therapy in this vulnerable population.

## References

[1] Egan BM Li J Hutchison FN: Hypertension in the United States, 1999 to 2012: Progress toward Healthy People 2020 goals. *Circulation.* 2014;130(19):1692–9. 10.1161/circulationaha.114.010676 25332288 PMC4221549

[2] Duprez DA: Systolic Hypertension in the Elderly: Addressing an Unmet Need. *Am J Med.* 2008;121(3):179–184.e3. 10.1016/j.amjmed.2007.10.027 18328297

[3] den Ouden MEM Schuurmans MJ Mueller-Schotte S: Do subclinical vascular abnormalities precede impaired physical ability and ADL disability? *Exp Gerontol.* 2014;58:1–7. 10.1016/j.exger.2014.06.002 24909353

[4] Ettinger WH Fried LP Harris T: Self-reported Causes of Physical Disability in Older People: The Cardiovascular Health Study. CHS Collaborative Research Group. *J Am Geriatr Soc.* 1994;42(10):1035–44. 10.1111/j.1532-5415.1994.tb06206.x 7930326

[5] Ezzati M Lopez AD Rodgers A: Selected major risk factors and global and regional burden of disease. *Lancet.* 2002;360(9343):1347–60. 10.1016/S0140-6736(02)11403-6 12423980

[6] Ferrucci L Guralnik JM Pahor M: Hospital diagnoses, Medicare charges, and nursing home admissions in the year when older persons become severely disabled. *JAMA.* 1997;277(9):728–34. 10.1001/jama.1997.03540330050034 9042845

[7] https://www.census.gov/content/dam/Census/library/publications/2016/demo/p95-16-1.pdf. https://www.census.gov/content/dam/Census/library/publications/2016/demo/p95-16-1.pdf.

[8] Moraes AAI Baena CP Muka T: Achieved systolic blood pressure in older people: A systematic review and meta-analysis. *BMC Geriatr.* 2017;17(1):279. 10.1186/s12877-017-0672-4 29207946 PMC5717809

[9] Prevention of stroke by antihypertensive drug treatment in older persons with isolated systolic hypertension. Final results of the Systolic Hypertension in the Elderly Program (SHEP). SHEP Cooperative Research Group. *JAMA.* 1991;265(24):3255–64. 10.1001/jama.1991.03460240051027 2046107

[10] Beckett NS Peters R Fletcher AE: Treatment of hypertension in patients 80 years of age or older. *N Engl J Med.* 2008;358(18):1887–98. 10.1056/NEJMoa0801369 18378519

[11] Liu L Wang JG Gong L: Comparison of Active Treatment and Placebo in Older Chinese Patients With Isolated Systolic Hypertension. Systolic Hypertension in China (Syst-China) Collaborative Group. *J Hypertens.* 1998;16(12 Pt 1):1823–9. 10.1097/00004872-199816120-00016 9869017

[12] Staessen JA Fagard R Thijs L: Randomised Double-Blind Comparison of Placebo and Active Treatment for Older Patients With Isolated Systolic Hypertension. The Systolic Hypertension in Europe (Syst-Eur) Trial Investigators. *Lancet.* 1997;350(9080):757–64. 10.1016/s0140-6736(97)05381-6 9297994

[13] Bavishi C Bangalore S Messerli FH: Outcomes of Intensive Blood Pressure Lowering in Older Hypertensive Patients. *J Am Coll Cardiol.* 2017;69(5):486–93. 10.1016/j.jacc.2016.10.077 28153104

[14] Amery A Birkenhäger W Brixko P: Mortality and morbidity results from the European Working Party on High Blood Pressure in the Elderly trial. *Lancet.* 1985;1(8442):1349–54. 10.1016/s0140-6736(85)91783-0 2861311

[15] Dahlöf B Hansson L Lindholm LH: Morbidity and mortality in the Swedish Trial in Old Patients with Hypertension (STOP-Hypertension). *Lancet.* 1991;338(8778):1281–5. 10.1016/0140-6736(91)92589-t 1682683

[16] Ogihara T Saruta T Rakugi H: Target blood pressure for treatment of isolated systolic hypertension in the elderly: Valsartan in elderly isolated systolic hypertension study. *Hypertension.* 2010;56(2):196–202. 10.1161/HYPERTENSIONAHA.109.146035 20530299

[17] Williamson JD Supiano MA Applegate WB: Intensive vs Standard Blood Pressure Control and Cardiovascular Disease Outcomes in Adults Aged ≥75 Years: A Randomized Clinical Trial. *JAMA.* 2016;315(24):2673–82. 10.1001/jama.2016.7050 27195814 PMC4988796

[18] Gottesman RF Albert MS Alonso A: Associations Between Midlife Vascular Risk Factors and 25-Year Incident Dementia in the Atherosclerosis Risk in Communities (ARIC) Cohort. *JAMA Neurol.* 2017;74(10):1246–54. 10.1001/jamaneurol.2017.1658 28783817 PMC5710244

[19] Supiano MA Williamson JD: Applying the Systolic Blood Pressure Intervention Trial Results to Older Adults. *J Am Geriatr Soc.* 2017;65(1):16–21. 10.1111/jgs.14681 28111758

[20] Tzourio C Anderson C Chapman N: Effects of blood pressure lowering with perindopril and indapamide therapy on dementia and cognitive decline in patients with cerebrovascular disease. *Arch Intern Med.* 2003;163(9):1069–75. 10.1001/archinte.163.9.1069 12742805

[21] Forette F Seux ML Staessen JA: The prevention of dementia with antihypertensive treatment: New evidence from the Systolic Hypertension in Europe (Syst-Eur) study. *Arch Intern Med.* 2002;162(18):2046–52. 10.1001/archinte.162.18.2046 12374512

[22] Forette F Seux ML Staessen JA: Prevention of dementia in randomised double-blind placebo-controlled Systolic Hypertension in Europe (Syst-Eur) trial. *Lancet.* 1998;352(9137):1347–51. 10.1016/s0140-6736(98)03086-4 9802273

[23] van Charante EPM Richard E Eurelings LS: Effectiveness of a 6-year multidomain vascular care intervention to prevent dementia (preDIVA): A cluster-randomised controlled trial. *Lancet.* 2016;388(10046):797–805. 10.1016/S0140-6736(16)30950-3 27474376

[24] Williamson JD Launer LJ Bryan RN: Cognitive function and brain structure in persons with type 2 diabetes mellitus after intensive lowering of blood pressure and lipid levels: A randomized clinical trial. *JAMA Intern Med.* 2014;174(3):324–33. 10.1001/jamainternmed.2013.13656 24493100 PMC4423790

[25] Prince MJ Bird AS Blizard RA: Is the cognitive function of older patients affected by antihypertensive treatment? Results from 54 months of the Medical Research Council's treatment trial of hypertension in older adults. *BMJ.* 1996;312(7034):801–5. 10.1136/bmj.312.7034.801 8608285 PMC2350726

[26] SPRINT MIND Investigators for the SPRINT Research Group, Williamson JD Pajewski NM: Effect of Intensive vs Standard Blood Pressure Control on Probable Dementia: A Randomized Clinical Trial. *JAMA.* 2019;321(6):553–61. 10.1001/jama.2018.21442 30688979 PMC6439590

[27] Divisón-Garrote JA Ruilope LM de La Sierra A: Magnitude of Hypotension Based on Office and Ambulatory Blood Pressure Monitoring: Results From a Cohort of 5066 Treated Hypertensive Patients Aged 80 Years and Older. *J Am Med Dir Assoc.* 2017;18(5):452.e1–452.e6. 10.1016/j.jamda.2017.01.015 28246017

[28] Tinetti ME Han L Lee DSH: Antihypertensive medications and serious fall injuries in a nationally representative sample of older adults. *JAMA Intern Med.* 2014;174(4):588–95. 10.1001/jamainternmed.2013.14764 24567036 PMC4136657

[29] Whelton PK Carey RM Aronow WS: 2017 ACC/AHA/AAPA/ABC/ACPM/AGS/APhA/ASH/ASPC/NMA/PCNA Guideline for the Prevention, Detection, Evaluation, and Management of High Blood Pressure in Adults: A Report of the American College of Cardiology/American Heart Association Task Force on Clinical Practice Guidelines. *J Am Coll Cardiol.* 2018;71(19):e127-e248. 10.1016/j.jacc.2017.11.006 29146535

[30] Bakris G Ali W Parati G: ACC/AHA Versus ESC/ESH on Hypertension Guidelines: JACC Guideline Comparison. *J Am Coll Cardiol.* 2019;73(23):3018–26. 10.1016/j.jacc.2019.03.507 31196460

[31] Correction: Benefits and Harms of Intensive Blood Pressure Treatment in Adults Aged 60 Years or Older. *Ann Intern Med.* 2018;168(7):529. 10.7326/L18-0077 29610912

[32] Hansson L Zanchetti A Carruthers SG: Effects of Intensive Blood-Pressure Lowering and Low-Dose Aspirin in Patients With Hypertension: Principal Results of the Hypertension Optimal Treatment (HOT) Randomised Trial. HOT Study Group. *Lancet.* 1998;351(9118):1755–62. 10.1016/s0140-6736(98)04311-6 9635947

[33] Wei Y Jin Z Shen G: Effects of intensive antihypertensive treatment on Chinese hypertensive patients older than 70 years. *J Clin Hypertens (Greenwich).* 2013;15(6):420–7. 10.1111/jch.12094 23730991 PMC8033887

[34] Weiss J Freeman M Low A: Benefits and Harms of Intensive Blood Pressure Treatment in Adults Aged 60 Years or Older: A Systematic Review and Meta-analysis. *Ann Intern Med.* 2017;166(6):419–429. 10.7326/M16-1754 28114673

[35] Warwick J Falaschetti E Rockwood K: No evidence that frailty modifies the positive impact of antihypertensive treatment in very elderly people: An investigation of the impact of frailty upon treatment effect in the HYpertension in the Very Elderly Trial (HYVET) study, a double-blind, placebo-controlled study of antihypertensives in people with hypertension aged 80 and over. *BMC Med.* 2015;13:78. 10.1186/s12916-015-0328-1 25880068 PMC4404571

[36] Materson BJ Garcia-Estrada M Preston RA: Hypertension in the frail elderly. *J Am Soc Hypertens.* 2016;10(6):536–41. 10.1016/j.jash.2016.03.187 27118485

[37] Muller M Smulders YM de Leeuw PW: Treatment of hypertension in the oldest old: A critical role for frailty? *Hypertension.* 2014;63(3):433–41. 10.1161/HYPERTENSIONAHA.113.00911 24324042

[38] Benetos A Bulpitt CJ Petrovic M: An Expert Opinion From the European Society of Hypertension–European Union Geriatric Medicine Society Working Group on the Management of Hypertension in Very Old Frail Subjects. *Hypertension.* 2016;67(5):820–5. 10.1161/HYPERTENSIONAHA.115.07020 26975708

[39] Onder G Landi F Fusco D: Recommendations to prescribe in complex older adults: Results of the CRIteria to assess appropriate Medication use among Elderly complex patients (CRIME) project. *Drugs Aging.* 2014;31(1):33–45. 10.1007/s40266-013-0134-4 24234805

[40] Rostami H Khayyatzadeh SS Tavakoli H: The relationship between adherence to a Dietary Approach to Stop Hypertension (DASH) dietary pattern and insomnia. *BMC Psychiatry.* 2019;19(1):S7. 10.1186/s12888-019-2220-6 31362734 PMC6668174

[41] Thomopoulos C Parati G Zanchetti A: Effects of blood pressure lowering on outcome incidence in hypertension: 4. Effects of various classes of antihypertensive drugs--overview and meta-analyses. *J Hypertens.* 2015;33(2):195–211. 10.1097/HJH.0000000000000447 25485720

[42] Yusuf S Sleight P Pogue J: Effects of an angiotensin-converting-enzyme inhibitor, ramipril, on cardiovascular events in high-risk patients. *N Engl J Med.* 2000;342(3):145–53. 10.1056/NEJM200001203420301 10639539

[43] Lithell H Hansson L Skoog I: The Study on Cognition and Prognosis in the Elderly (SCOPE): Principal results of a randomized double-blind intervention trial. *J Hypertens.* 2003;21(5):875–86. 10.1097/00004872-200305000-00011 12714861

[44] Aronow WS Fleg JL Pepine CJ: ACCF/AHA 2011 Expert Consensus Document on Hypertension in the Elderly: A Report of the American College of Cardiology Foundation Task Force on Clinical Expert Consensus Documents *Developed in Collaboration With the American Academy of Neurology, American Geriatrics Society, American Society for Preventive Cardiology, American Society of Hypertension, American Society of Nephrology, Association of Black Cardiologists, and European Society of Hypertension*. *J Am Coll Cardiol.* 2011;57(20):2037–114. 10.1016/j.jacc.2011.01.008 21524875

[45] Mediavilla García JD Jaén Águila F Fernández Torres C: Ambulatory blood pressure monitoring in the elderly. *Int J Hypertens.* 2012;2012:548286. 10.1155/2012/548286 22229085 PMC3249829

[46] Moonen JEF Foster-Dingley JC de Ruijter W: Effect of discontinuation of antihypertensive medication on orthostatic hypotension in older persons with mild cognitive impairment: The DANTE Study Leiden. *Age Ageing.* 2016;45(2):249–55. 10.1093/ageing/afv199 26758532

